# Interaction of Sirtuin 1 (SIRT1) candidate longevity gene and particulate matter (PM2.5) on all-cause mortality: a longitudinal cohort study in China

**DOI:** 10.1186/s12940-021-00718-x

**Published:** 2021-03-14

**Authors:** Yao Yao, Linxin Liu, Guang Guo, Yi Zeng, John S. Ji

**Affiliations:** 1 Center for Healthy Aging and Development Studies, National School of Development, Peking University, Beijing, China; 2 Environmental Research Center, Duke Kunshan University, 22 Address: No. 8 Duke Avenue, Kunshan, 215316 Jiangsu China; 3 Department of Sociology, Carolina Population Center, and Carolina Center for Genome Sciences, University of North Carolina at Chapel Hill, Chapel Hill, NC USA; 4 Center for the Study of Aging and Human Development, Duke Medical School, Durham, NC USA; 5 Nicholas School of the Environment, Duke University, Durham, NC USA

**Keywords:** Longevity gene, Air pollution, Sex difference, CLHLS

## Abstract

**Background:**

The *SIRT1* gene was associated with the lifespan in several organisms through inflammatory and oxidative stress pathways. Long-term air particulate matter (PM) is detrimental to health through the same pathways.

**Methods:**

We used the Chinese Longitudinal Healthy Longevity Survey (CLHLS) to investigate whether there is a gene-environment (G × E) interaction of *SIRT1* and air pollution on mortality in an older cohort in China. Among 7083 participants with a mean age of 81.1 years, we genotyped nine *SIRT1* alleles for each participant and assessed PM_2.5_ concentration using 3-year average concentrations around each participant’s residence. We used Cox-proportional hazards models to estimate the independent and joint effects of *SIRT1* polymorphisms and PM_2.5_ exposure on all-cause mortality, adjusting for a set of confounders.

**Results:**

There were 2843 deaths over 42,852 person-years. The mortality hazard ratio (HR) and 95% confidence interval (CI) for each 10 μg/m^3^ increase in PM_2·5_ was 1.08 (1.05–1.11); for *SIRT1*_391 was 0.77 (0.61, 0.98) in the recessive model after adjustment. In stratified analyses, participants carrying two *SIRT1*_391 minor alleles had a significantly higher HR for each 10 μg/m^3^ increase in PM_2.5_ than those carrying zero minor alleles (1.323 (95% CI: 1.088, 1.610) vs. 1.062 (1.028, 1.096) p for interaction = 0.03). Moreover, the interaction of *SIRT1* and air pollution on mortality is significant among women but not among men. We did not see significant relationships for *SIRT1*_366, *SIRT1*_773, and *SIRT1*_720.

**Conclusion:**

We found a gene-environment interaction of *SIRT1* and air pollution on mortality, future experimental studies are warranted to depict the mechanism observed in this study.

**Supplementary Information:**

The online version contains supplementary material available at 10.1186/s12940-021-00718-x.

## Introduction

Sirtuin 1 (*SIRT1)* is a gene well-documented to be associated with aging and longevity [1]. The *SIRT1* gene encodes proteins related to mammalian nicotinamide adenine dinucleotide (NAD+) dependent histone deacetylase. The sirtuin pathway is implicated in regulating lifespan in many model organisms such as yeast, *Caenorhabditis elegans*, and rodents through several biopathways, including anti-inflammation, regulation of metabolism, hypoxic responses, and circadian rhythms [2, 3]. In humans, *SIRT1* improves healthy aging and affects human life expectancy through its protective role in various biological processes related to age-related diseases ranging from metabolic disorders, cellular senescence, cardiac aging, oxidative stress, neurodegeneration, inflammatory signaling, and placental cell survival [1, 4]. A chronic, low-grade inflammation level characterizes human aging. Ambient air pollution is involved in developing degenerative diseases inclusive of aging arteries and brains through shared inflammatory pathways [5]. Biologically, *SIRT1* can modulate inflammatory genes such as *NF-κB* and *NLRP3* and further leads to delayed onset of age-related symptoms and pathologies [6, 7]. To date, quite a few observational studies linked *SIRT1* (i.e., SNP rs7896005, rs12778366, rs4746720) to long-term survival and longevity in the human populations, though these findings were restricted by either small population size or a cross-sectional cohort study design [8–11].

Air pollution is a significant detrimental environmental risk to human health, attributing to one in every nine deaths annually, and pollution mitigation is a top priority in the UN sustainable development goal (SDG) agenda [12]. The disease burden due to air pollution in many places is likely to continue to exacerbate, with many epidemiological studies continuing to document morbidities and mortalities [13, 14]. According to an analysis of the trend of air pollution in Global Burden of Disease Study, exposure to ambient fine particulate matter (PM_2.5_: particulate matter (PM) with aerodynamic diameters< 2.5 μm) is the fifth leading risk factor for death, accounting for 7.6% of total global deaths and 4.2% of global DALYs, with China accounting for a large share of these burdens [15]. China’s coal-based energy-intensive development path has led to a steep increase in emissions of PM_2.5_ and other pollutants [16], estimated to have led to 1.6 million deaths from heart and lung diseases or stroke, approximately accounting for one in six premature deaths in China [17]. Thus, the comprehensive health impact of air pollutions in Low- and Middle-income countries such as China remains one of the major health issues, and further attention, as well as joint approaches, are needed.

Increasingly, we have seen that air pollution does not affect everyone equally, with some populations more susceptible to its detrimental effects. Genetic susceptibility is likely to play a vital role in response to air pollution [18]. Inflammation and oxidative stress are documented to play a role in the mechanistic pathways, including nuclear factor kappa B cells (*NF-kB*) signaling, Krüppel-like Factor 2 (*Klf2*) mediated immune response, nuclear factor E2-realest factor 2 (*Nrf2*)-mediated oxidative stress response, NLR family pyrin domain containing 3 (*NLRP3*) inflammasome activation, glutathione metabolism, coagulation system, endogenous reactive oxygen species (ROS) production, and other cytokines signaling, between air pollution exposures and adverse health outcome including mortality [19]. Notably, biological studies indicated that *SIRT1* can be modulated through most of those pathways, including *NF-kB* [20]. *Klf2* [21], *Nrf2* [22], *NLRP3* [7], and ROS [23]. Additionally, previous experimental studies observed that air pollution and *SIRT1* have an interactive effect on pulmonary diseases [24, 25], cardiovascular diseases [26]. Although the associations between exposure to air pollutants and the *SIRT1* gene were observed in in vitro studies, less effort has been put forth in the investigation on a population level, especially for the vulnerable older adults. Additionally, epidemiologic evidence and rodent model showed the effect of *SIRT1* on disease and longevity vary by inflammatory levels [11, 27] and can be a double-edged sword: lower levels of *SIRT1* (short term exposure of toxicants) accentuate acute inflammation-related autotoxicity by increasing *NFκB RelA/p65* activity, but prolonged upgrading in *SIRT1* in later inflammation are associated with immunosuppression and increased mortality [28]. Given the *SIRT1* and PM_2.5_ shared several common biological pathways containing inflammation and oxidative stress on mortality and are tended to interplay with each other [26, 29], we hypothesized a potential synergistic effect between PM_2.5_ exposure and *SIRT1* polymorphisms on mortality.

To test our hypotheses, we used a nationally representative cohort of individuals aged 65 and older from the Chinese Longitudinal Healthy Longevity Study (CLHLS). First, we aim to estimate the independent and joint effects of *SIRT1* polymorphisms and PM_2.5_ exposure on all-cause mortality. Second, we take advantage of the sample size to study the interaction effect of *SIRT1* and air pollution on mortality. Considering the “male-female health-survival paradox” in longevity research, there are marked sex differences in genetic associations with longevity; thus, we also assessed the three-way interaction by sex.

## Method

### Study population

**Fig. 1:**
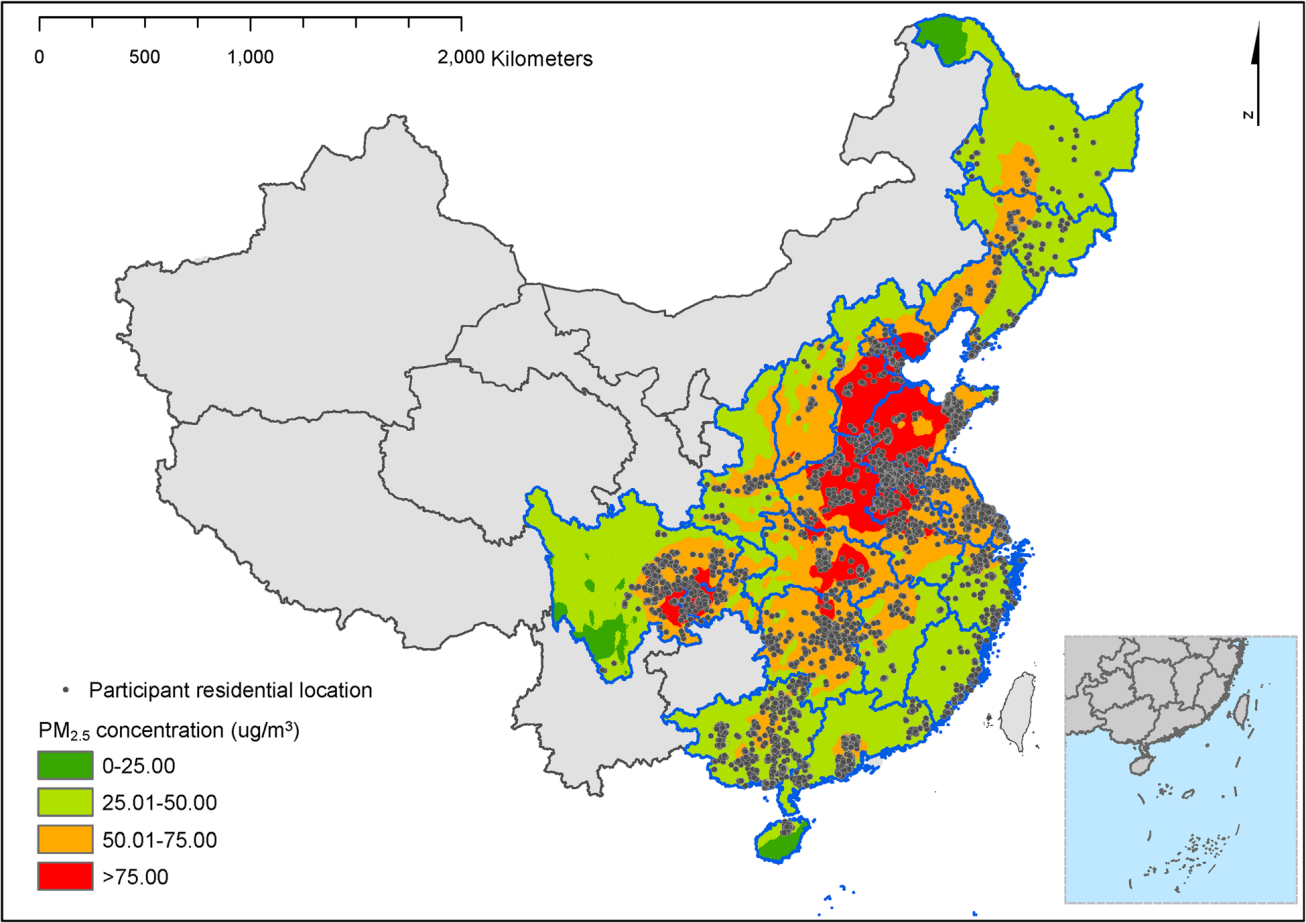
Distribution of the study population from participants of the Chinese Longitudinal Healthy Longevity Survey (CLHLS)

We used data from the Chinese Longitudinal Healthy Longevity Study (CLHLS), which are publicly available from Peking University Open Research Data (https://opendata.pku.edu.cn/dataverse/CHADS). The baseline and follow-up surveys were conducted in 1998, 2000, 2002, 2005, 2008–2009, 2011–2012, and 2014 in a randomly selected half of the counties and cities in 23 out of 31 provinces in China. The study was the first national longitudinal survey on the determinants of healthy aging among the oldest old individuals in China. Details of descriptions of the CLHLS including the rationale and design have been described previously [30]. With 631 cities and counties randomly selected as the sample sites, the population in 23 selected provinces represents 85% of the total population in China (Fig. 1). CLHLS was approved by the Institutional Review Board, Duke University (Pro00062871), and the Biomedical Ethics Committee, Peking University (IRB00001052–13074). All participants or their legal representatives signed written consent forms to participate in the baseline and follow-up surveys. This study followed the Strengthening the Reporting of Observational Studies in Epidemiology (STROBE) reporting guidelines.

There was a total of 34,394 participants in 5 waves of CLHLS, recruited from 2000 to 2011. We excluded 25,220 participants without available SIRT1 genotypic data or did not meet the selection criteria of sample filtering, 545 participants aged 64 or younger, 212 participants with missing PM_2.5_ values, 567 participants were not of Han ethnicity (according to the ID card or household registry) or having a missing value in covariates; 767 participants lost to follow-up at the first follow-up survey. The samples were required to meet 3 selection criteria: (1) a genotype calling rate > 90%; (2) no existing population stratification according to multidimensional scaling (MDS) procedure implemented in PLINK v1.07, based on which individuals deviating from the main population cluster were removed; and (3) no inclusion of duplicates or first-degree relatives when evaluating pairwise through identity by descent (IBD). Accordingly, the final sample that met inclusion criteria for this study was 7083 participants (Fig. S1). The sample consisted of 3677 women and 3406 men; 3272 participants were 65 to 79 years of age, 1840 were 80–89 years of age, 1305 were 90–99 years of age, and 667 were 100 years of age or older. To test the possibility of potential selection bias, gender, age, and residence were compared between participants who lost to follow-up (767 participants) or not (7083 participants) at the first follow-up survey; the significant difference for ages (86.0 vs 81.1) and residence (rural: 53.8% vs 66.7%) between two groups, while no significant difference for sex (female: 52.7% vs 51.9%).

### Assessment of PM_2.5_ exposure

We estimated ground-level concentrations of PM_2.5_ from the Atmospheric Composition Analysis Group based on participants’ residential addresses [31]. It combines remote sensing from National Aeronautics and Space Administration’s Moderate Resolution Imaging Spectroradiometer, Multiangle Imaging Spectroradiometers, and Sea-viewing Wide field-of-view Sensor satellite instruments; vertical profiles derived from the GEOS-Chem chemical transport model; and calibration to ground-based observations of PM_2.5_ using geographically weighted regression. Annual PM_2.5_ estimates were calculated from 2000 to 2014, at 1 km × 1 km spatial resolution, which was the longest and the highest resolution exposure dataset available [32, 33]. Additionally, our estimations were highly consistent with out-of-sample cross-validated concentrations from monitors (R^2^ = 0·81) and another exposure dataset in China (R^2^ = 0·79) [31]. A previous study found that the three-year average PM_2.5_ before death or the end of the study had the strongest association with mortality among old adults in China [32]. Therefore, we used a three-year average PM_2.5_ to reflect ambient air pollution in this study.

### The *SIRT1* genotypes

We customize an SNP chip containing 27,656 selected longevity and disease-related SNPs for targeted genotyping [34]. We selected candidate SNPs from the public database of the National Center for Biotechnology Information (NCBI; http://www-ncbi-nlm-nih-gov/gene/23411) to cover the *SIRT1* gene region in equally spaced intervals [35]. The minor allele frequencies (MAF) of the polymorphisms are required to be > 10% [36]. The selected and genotyped *SIRT1* SNPs were rs12778366 (promoter), rs3758391 (promoter), rs2273773 (exon), rs2236319 (intron), rs1885472 (intron), rs7069102 (intron), rs10823112 (intron), rs3818291 (intron), and rs4746720 (intron), which have covered major functional SNPs in *SIRT1* gene. Among the 9 SNPs, *SIRT1*_366 (rs12778366), *SIRT1*_391 (rs3758391), *SIRT1*_773 (rs2273773), and *SIRT1*_720 (rs4746720) were annotated and regarded as tagging SNPs due to the high linkage disequilibrium, which were used as proxies for the rest of 5 SNPs in the analysis (Table S1). The Hardy-Weinberg equilibrium of the 9 *SIRT1* SNPs was tested with the GENEPOP package (version 1.2). To determine the *SIRT1* carrier, we coded the genotype based on the minor allele number [37]. For each SNP, three different inheritance models (additive, dominant, and recessive models) were tested. In the additive model, the genotype that contains zero, one, or two copies of minor allele was coded as 0, 1 or 2; In the dominant model, the genotype that contains at least one copy of minor allele was coded as 1, otherwise, it was coded as 0; In the recessive model, the genotype that contains two copies of minor allele was coded as 1 and otherwise it was coded as 0.

### All-cause mortality

The primary outcome was all-cause mortality. Mortality information was obtained from the follow-up survey done in 2011 and 2014. The date of death would be validated by death certificates when available - otherwise, the close family member’s report was used.

### Covariates

The investigator chose covariates that may be potential confounders between exposures and outcomes or predictors of outcomes based on a prior knowledge and a causal framework. All self-reported information was collected through face-to-face home interviews by trained research staff members. Interviewees were encouraged to answer as many questions as possible. If they were unable to answer questions, a close family member or another proxy, such as a primary caregiver, provided answers. We included baseline age, gender, marital status, residence, education, occupation, smoking status, drinking status, physical activity, and the wave of the first interview as covariates. We classified marital status into two categories: currently married and living with spouse as married, and widowed/separated/divorced/never married/married but not living with spouse as not married. We divided residences into urban and rural areas based on governmental administrative categories. We used the schooling year to evaluate education level. We categorized the occupation into two groups: professional and technical personnel, governmental, institutional, or managerial personnel as non-manual, and agriculture, forest, animal husbandry, fishery worker, industrial worker, and others as manual. We divided the regular exercise, smoking, and alcohol drinking status into three categories: “Current”, “Former”, and “Never”. For example, participants were asked, “do you do exercise regularly at present (planned exercise like walking, playing balls, running and so on)?” and/or “did you do exercise regularly in the past?”. We defined the regular exercise status as “Current” for participants who answered “Yes” to the first question, “Former” for those who answered “No” to the first question and “Yes” to the second question, and “Never” for those who answered “No” to both two questions. We categorized the participants into two geographical regions: South China (Guangdong, Guangxi, Hainan, Chongqing, Sichuan, Anhui, Hubei, Fujian, Jiangxi, Jiangsu, Shanghai, and Zhejiang) and North China (Beijing, Shandong, Heilongjiang, Jilin, and Liaoning, Hebei, Henan, Shanxi, Tianjin, and Shaanxi).

### Statistical analysis

We used the cox proportional hazard model for every *SIRT1* SNP and three-year average PM_2.5_ (as continuous variables or category variables by quartiles) separately to evaluate their single effect on mortality. We added the interaction term of SNP and three-year average PM_2.5_ in the cox model to investigate the interaction of *SIRT1* and three-year average PM_2.5_. The genotype can be defined as low-risk genotype (G0) and high-risk genotype (G1) based on the genotypes’ hazard ratio for mortality risk. The three-year average PM_2.5_ was discretized into two categories as low PM_2.5_ exposure (E0) versus high PM_2.5_ exposure (E1) using the median of three-year average PM_2.5_ as the cut-off point. The gene effect in different environment exposure and environmental effect among participants with different genotypes can be evaluated by comparing the difference among the four categories (G0 × E0 stands for low-risk genotype under low PM2.5 exposure, G0 × E1 stands for low-risk genotype under high PM2.5 exposure, G1 × E0 stands for high-risk genotype under low PM2.5 exposure, and G1 × E1 stands for high-risk genotype under high PM2.5 exposure) of the combined term. To investigate the gender-specific G × E effect, we adopt an integrated statistical model of three-way interaction to assess varied effect magnitude in the hazard ratio of mortality between those who have different combinations of sex, *SIRT1* genotypes, and exposure to PM_2.5_ [38].

We measured the survival time in months from the first interview date to the recorded death date or last interview date. Our statistician adjusted all models for age, gender, marital status, residence area, education, occupation, smoking status, drinking status, and physical activity. We calculated hazard ratios (HRs) and 95% CIs to indicate the effect magnitude of *SIRT1* and PM_2.5_ on mortality. We analyzed for effect modification by potential modifier variables, then did stratified analyses by sex, urban or rural residence, financial status, smoking status, and North or South geographical regions.

We did additional analyses to verify the results. We compared baseline characteristics between the included participants and all participants in the five waves of CLHLS to assess the sample’s representativeness. We adjusted the regression models by using more informative covariates and by excluding covariates with missing values. We also plotted the geographical distribution of the included participants.

We used R 3.6.1 (R Foundation for Statistical Computing) and SAS university edition to perform all the analyses. All *p* values were from 2-sided tests, and results were deemed statistically significant at *p* < .05 for all analyses.

## Results

Among the 7083 participants, 48.1% were men, and 51.9% were women, with a mean (SD) age of 81.1 (11.5) years. More than half of the participants were illiterate (57.1%), residents in rural (66.7%), responded to no engage in regular exercise (64.5%), never smoked (64·0%), and never drank alcohol (68.5%) (Table S2). The mean 3-year PM_2.5_ was 51.0 μg/m^3^ (13.5), with populations in rural regions and southern regions experiencing lower ambient air pollution. Four tag SNPs (*SIRT1*_366, *SIRT1*_391, *SIRT1*_773, and *SIRT1*_720) were used to represent the 9 *SIRT1* SNPs as they had high Linkage Disequilibrium (r^2^ ≥ 0.975; Fig. S2). The distributions of the tag SNPs of *SIRT1* are similar across varied baseline characteristics, including sex, age, and education (Table S2), indicating *SIRT1* SNPs were randomly distributed across population characteristics. While participants who were older, illiterate, responded of no regular exercise, reside in northern China tended to live in areas with higher PM_2.5_ exposure (Table S2). The final sample has a lower average of age, more likely to be men and rural residents, had a higher proportion of currently married status, than those who were excluded; no significant difference has been detected regarding the education level, exercise habit, smoking and drinking status between the two groups (Table S3).

The mean follow-up time (SD) was 6.1 (3.5) years (range: 0 to 14 years). During the 42,852 person-years of follow-up, we saw 2843 mortality events (40.1%) between 2002 and 2014. The mortality rate was 6.6 per 100 person-years for our entire study population. In the additive model, *SIRT1*_391, *SIRT1*_472, and *SIRT1*_102 minor allele carriers had lower mortality than their counterparts. The recessive model showed similar results to the addictive model, yet no statistical significance was observed in the dominant model (Table 1). We also did not see a significant association of *SIRT1*_366, *SIRT1*_773, and *SIRT1*_720 with mortality.

**Table 1:** The association between *SIRT1* SNPs with mortality

Minor allele copy number	n	HR (95% CI)	***p*** value
*SIRT1*_366 (*n* = 7055)			
Additive model			
0	5281	Ref	
1	1635	1.050 (0.963,1.146)	0.269
2	139	1.209 (0.905,1.615)	0.199
Dominant model			
0	5281	Ref	
1 or 2	1774	1.059 (0.973,1.153)	0.182
Recessive model			
0 or 1	6916	Ref	
2	139	1.195 (0.895,1.595)	0.227
*SIRT1*_391 (*n* = 7077)			
Additive model			
0	5006	Ref	
1	1887	1.023 (0.942,1.111)	0.589
2	184	0.769 (0.603,0.981)	0.035
Dominant model			
0	5006	Ref	
1 or 2	2071	0.997 (0.92,1.081)	0.947
Recessive model			
0 or 1	6893	Ref	
2	184	0.764 (0.599,0.974)	0.030
*SIRT1*_773 (*n* = 7071)			
Additive model			
0	3795	Ref	
1	2743	0.993 (0.919,1.072)	0.854
2	533	0.960 (0.825,1.117)	0.597
Dominant model			
0	3795	Ref	
1 or 2	3276	0.988 (0.918,1.064)	0.748
Recessive model			
0 or 1	6538	Ref	
2	533	0.963 (0.831,1.116)	0.616
*SIRT1*_720 (*n* = 7046)			
Additive model			
0	2292	Ref	
1	3383	1.045 (0.961,1.137)	0.302
2	1371	1.013 (0.910,1.127)	0.814
Dominant model			
0	2292	Ref	
1 or 2	4754	1.036 (0.957,1.121)	0.383
Recessive model			
0 or 1	5675	Ref	
2	1371	0.986 (0.898,1.083)	0.774

Note: All models were adjusted for age at baseline, sex, education, marriage, occupation, residence, exercise, smoking, and alcohol consumption

In the fully adjusted model, the all-cause mortality hazard ratio (HR) and 95% CI for each 10 μg/m^3^ increase in PM_2.5_ was 1.082 (1.053, 1.111). We also conducted our analysis by quartiles to account for a possible nonlinear dose-response relationship between PM_2.5_ exposure and risk of longevity. Compared with the participants who resided in areas with the lowest quartile of PM_2.5_, the HR of mortality for the 2nd quartile, 3rd quartile, and 4th quartile were 1.203 (1.078, 1.343), 1.431 (1.287, 1.593), 1.297 (1.167, 1.442), respectively (Table 2). In the stratified analyses by tags of *SIRT1* genotypes, participants who carry zero or one *SIRT1*_391 minor allele had an HR of 1.078 (95% CI: 1.049,1.107) on mortality for each 10 μg/m^3^ increase in PM_2.5_, while the HR was 1.336 (95% CI: 1.079, 1.653) in those who carry two *SIRT1*_391 minor alleles (data not shown).

**Table 2:** The association between three-year average PM_2.5_ with mortality

PM_**2.5**_	n	HR (95% CI)	***p*** value
Quartiles of PM_2.5_			
Quartile 1	1786	1.00 (reference)	/
Quartile 2	1761	1.203 (1.078, 1.343)	0.000939
Quartile 3	1765	1.431 (1.287, 1.593)	4.42E-11
Quartile 4	1771	1.297 (1.167, 1.442)	1.37E-06
10-μg/m^3^ change in PM_2.5_	/	1.082 (1.053, 1.111)	1.20E-08

Note: All models were adjusted for age at baseline, sex, education, marriage, occupation, residence, exercise, smoking, and alcohol consumption

According to an addictive model with interaction terms of *SIRT1* SNPs and PM_2.5_, the detrimental effect of PM_2.5_ exposure on mortality for the participants carrying two *SIRT1*_391 minor alleles was stronger than those carrying zero alleles. We saw similar results in the *SIRT1*_472 and *SIRT1*_102, of which were in LD with *SIRT1*_391. For instance, the test for the interaction between *SIRT1*_391 and PM_2.5_ in additive models revealed a significant gene-environment interaction (*p* for interaction = 0.03). The HR of 10-μg/m^3^ 3-year average PM_2.5_ on mortality for the participants carrying two *SIRT1*_391 minor alleles was e^(0.06 + 0.220)^ = 1.323 (95% CI: 1.088, 1.610) and for those carrying zero minor allele would be e^0.06^ = 1.062 (1.028, 1.096) (Table 3). We also tested the interaction effect of *SIRT1* SNPs and PM_2.5_ in the recessive model, and the results are not meaningfully different from findings based on the additive model (Table S4). As for the dominant model, we found there was a difference of PM_2.5_ in mortality between participants carrying one or two alleles and those carrying zero alleles (Table S5).

**Table 3:** The interaction between PM_2.5_ and *SIRT1* SNPs (additive model) on mortality

Additive model	Model without interaction term		Model with interaction term		
	HR (95% CI)	*p* value	*β*	SE	*p* value
Carrying *SIRT1*_366 minor allele status					
0 copy	Ref	/	/	/	/
1 copy	1.059 (0.970,1.155)	0.200	0.248	0.171	0.147
2 copies	1.245 (0.931,1.663)	0.139	0.011	0.545	0.984
10-μg/m^3^ unit of PM_2.5_	1.083 (1.054,1.113)	7.04E-09	0.088	0.016	3.50E-08
Interaction term					
zero *SIRT1*_366 minor allele×PM_2.5_	/	/	/	/	/
one *SIRT1*_366 minor allele×PM_2.5_	/	/	−0.037	0.032	0.249
two *SIRT1*_366 minor alleles×PM_2.5_	/	/	0.043	0.105	0.685
Carrying *SIRT1_391* minor allele status					
0 copy	Ref	/	/	/	/
1 copy	1.026 (0.944,1.114)	0.548	−0.255	0.167	0.127
2 copies	0.768 (0.602,0.980)	0.034	−1.452	0.577	0.012
10-μg/m^3^ unit of PM_2.5_	1.082 (1.053,1.112)	1.07E-08	0.06	0.016	2.64E-04
Interaction term					
zero *SIRT1*_391 minor allele×PM_2.5_	/	/	/	/	/
one *SIRT1*_391 minor allele×PM_2.5_	/	/	0.054	0.031	0.081
two *SIRT1*_391 minor alleles×PM_2.5_	/	/	0.220	0.101	0.030
Carrying *SIRT1_773* minor allele status					
0 copy	Ref	/	/	/	/
1 copy	1.001 (0.926,1.081)	0.986	0.109	0.153	0.478
2 copies	0.951 (0.817,1.106)	0.513	−0.379	0.318	0.234
10-μg/m^3^ unit of PM_2.5_	1.082 (1.053,1.112)	1.19E-08	0.083	0.019	1.33E-05
Interaction term					
zero *SIRT1*_773 minor allele×PM_2.5_	/	/	/	/	/
one *SIRT1*_773 minor allele×PM_2.5_	/	/	−0.021	0.029	0.464
two *SIRT1*_773 minor alleles×PM_2.5_	/	/	0.06	0.056	0.286
Carrying *SIRT1_720* minor allele status					
0 copy	Ref	/	/	/	/
1 copy	1.044 (0.960,1.135)	0.319	0.381	0.168	0.023
2 copies	1.001 (0.899,1.113)	0.992	0.201	0.223	0.366
10-μg/m3 unit of PM_2.5_	1.081 (1.052,1.111)	1.93E-08	0.117	0.024	1.45E-06
Interaction term					
zero *SIRT1*_720 minor allele×PM_2.5_	/	/	/	/	/
one *SIRT1*_720 minor allele×PM_2.5_	/	/	−0.065	0.031	0.036
two *SIRT1*_720 minor alleles× PM_2.5_	/	/	−0.038	0.041	0.345

Note: All models were adjusted for age at baseline, sex, education, marriage, occupation, residence, exercise, smoking, and alcohol consumption

**Fig. 2:**
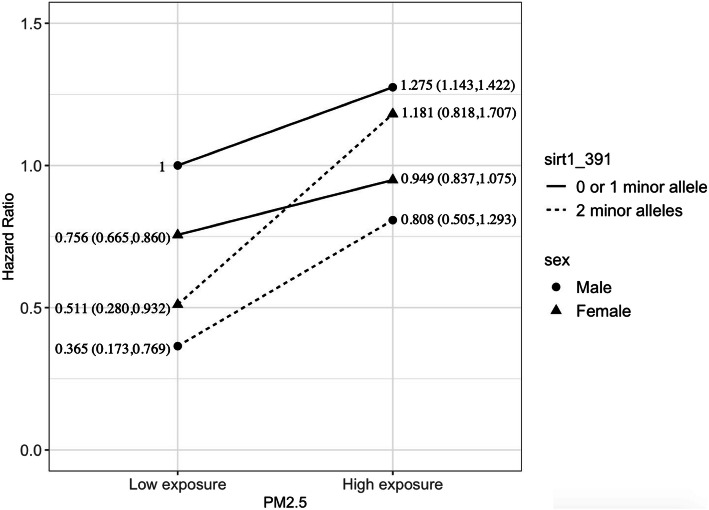
The sex-specific interaction analysis on PM_2.5_, *SIRT1*_391 and mortality. Note: The male participants without two *SIRT1_*391 minor allele copies and with low exposure of PM_2.5_ was regarded as the reference group. The model adjusted for age at baseline, sex, education, marriage, occupation, residence, exercise, smoking, and alcohol consumption

Further, we conducted a three-way interaction analysis to examine whether there is a sex difference in the effect of G × E interaction. Figure 2 demonstrates exposure to higher PM_2.5_ does not affect mortality significantly in males and females who carry zero or one *SIRT1*_391 allele, but PM_2.5_ exposure substantially increases the mortality risk among male and female carriers of two *SIRT1*_391 alleles. It is noted that among the participants with exposure to the high concentration of PM_2.5_, females carry two *SIRT1*_391 alleles have a significant excess risk of mortality than those with zero or one *SIRT1*_391 allele; but the interaction is not significant among male participants. Specifically, in females who were carrying two *SIRT1*_391, exposure to a higher level of PM_2.5_ is associated with a 47.7% higher risk of mortality, while the effect size is 16.8% in men.

## Discussion

Our study indicated/replicated the detrimental effect of air pollution on all-cause mortality. We found a protective effect of some alleles of *SIRT1* on all-cause mortality, in concurrence with several prior animal studies and population studies. Our study’s added evidence is finding an interaction between PM_2.5_ induced premature mortality and carriers of *SIRT1*_391, *SIRT1*_472, and *SIRT1*_102 genotypes, which carriers of 2 alleles of *SIRT1*_391 can counteract the detrimental effect of PM_2.5_ and reduce 26.1% risk of premature mortality. Although none of the associations between SNPs and mortality would be statistically significant after the most stringent Bonferroni correction (α = 0.05/4 ≈ 0.0125), we used candidate SNPs tested in previous studies instead of random SNPs so that the need for stringent correction may not be necessitated. Our study used a large old cohort to elucidate better evidence of several cellular and molecular pathways studies on the protective role of *SIRT1*. Prior studies indicate *SIRT1* is involved in the pathway of PM-induced airway inflammation with the activation of *SIRT1* to prevent airway disorders [24, 25]. On the cellular and molecular side, using in vivo models of airway inflammation and in vitro culture of human bronchial epithelial (HBE) cells exposed to PM_2.5_ and resveratrol (*SIRT1* activator), *SIRT1* expression was decreased in HBE cells and lung tissues after PM_2.5_ exposure, suggesting that *SIRT1* is involved in the pathogenesis of PM-induced airway inflammation [29]. Second, a plethora of animal studies investigating PM_2.5_ has documented pathways of inducing oxidative stress to trigger inflammation and thrombosis [39]. It was hypothesized that *SIRT1* as a member of class III histone deacetylase, controls lung inflammation and coagulation after PM_2.5_ exposure. *SIRT1* knock-out mice exhibited aggravated lung vascular leakage and inflammation after PM_2.5_ exposure, correlated with increased *NF-κB* acetylation and activation. This indicates that SIRT1 functions as a suppressor of coagulation after particulate matter exposure [29]. Further molecular evidence also implicates the role of *SIRT1* in protein/histone deacetylase and as a protective factor of the development of pulmonary emphysema [40]. PM_2.5_ exposure in animal models was also demonstrated to introduce the repression activity of *SIRT1* in the mice liver [41].

Our study builds on these animal models and is the most extensive population-based study on the role of *SIRT1* in air pollution-induced mortality. Previously, a series of genes involved in oxidative stress and inflammatory pathways were studied for interaction with air pollutants, including *GSTM1*, *GSTP1*, *NQO1*, and *TNF.* There were both positive and null findings [42]. Studies indicate genetic susceptibility is likely to play a role in response to air pollution, especially on cardiovascular and respiratory outcomes [29]. A recent review indicated that *SIRT1* plays in toxicological damage caused by environmental toxicants such as PM_2.5_, the PM-induced injury affect *SIRT1* expression, which then affects the expression and activity of downstream proteins, resulting in toxic damage [43]. In addition, one molecular biology finding suggested that PM_2.5_ can upregulate MicroRNA-146a-3p and induces inflammatory macrophage polarization by targeting *SIRT1* [44]. A recent population study also found that exposure to long-term air pollution, even at a low level, can alter gene expression and, in turn to impact an individual’s health [18]. Various studies have indicated that aging and lifespan are modulated by genetic and environmental factors as well as their interactions. Extant basic and translational studies have reported the interaction effects of air pollution and *SIRT1* on the incidence or progression of pulmonary diseases [25, 29, 45] and cardiovascular diseases [26]. As we do not have a complete understanding of the synergistic health effects of air pollution and *SIRT1* in humans, and prior studies are mainly focused on animal models, our finding of an interactive effect needs validation.

We also observe significant gender differentials in the interaction effect of PM_2.5_ and *SIRT1* genotypes on mortality. The association of PM_2.5_ exposure with a reversal of the negative effects of carrying two *SIRT1*_391 alleles on mortality is much stronger in females than in males. In females who were carrying two *SIRT1*_391, exposure to a higher level of PM_2.5_ is associated with a 47.7% higher risk of mortality, while the effect size is 16.8% in men. Underlying mechanisms of gender difference may be due to sex hormones and distinct innate immune systems [46]. First, estrogen can induce downregulation of *SIRT1*, which in turn reduces the anti-oxidative anti-inflammatory effect on PM-induced toxicants [47]. Although production of estrogen declined dramatically in females after menopause status, the estrogen remained at moderately low level. *SIRT1*, possibly through the *AKT* and *ERK* signaling pathways, plays a crucial role in estrogen in protecting arteries from senescence and atherosclerosis [48]. Resveratrol also functions as a suppressor of PM-induced inflammatory signaling pathways by inhibiting COX-2/PGE2 expression [49]. Second, sex-specific analyses of longevity gene indicate “the innate immune system in men and of the tryptophan and *PGC-1* pathways in the regulation of immune-related pathways in women suggests that women and men have different approaches for longevity, in which the *SIRT1* deacetylates *PGC-1α* and enhances *PGC-1α* activity, insufficient NAD^+^ availability and *SIRT1* enzymatic activity may be contributing factors [50]. Moreover, another sex-specific study on the human heart reveals a female sex-specific downregulation of *SIRT1* and *SIRT3* in aged hearts, as well as a decline in mitochondrial anti-oxidative defense and a pro-inflammatory shift in old female hearts but not in male hearts. The ex-specific downgrade of *SIRT1* in females than in males might predispose females to a more susceptible condition when exposed to PM-derived toxicants [51].

Our study has several limitations. The biggest limitation is that our cohort did not ascertain reliable cause-specific mortality, which means we could not determine the possible pathways of airway-induced mortality. Second, we used satellite-derived residential area-level PM_2.5_ measurement, which has been shown to be reliable but may not be as precise as ground-level monitors and personal monitors. However, we do not expect differential misclassification that biases our effect estimates. In addition, we used a 3-year average PM_2.5_, so we can only study long-term air pollution difference. Third, our study did not have biomarkers or dietary determinants of nicotinamide mononucleotide (NMN), an NAD+ precursor, to assess *SIRT1* activation activity; however, we do not expect these factors to varying with air pollution levels. Lastly, we also did not have biomarker data or clinical diagnosis of pulmonary function of the participants to ascertain differences based on *SIRT1* genotype status. Thus, our study cannot distinguish the possible mechanism of the interaction between *SIRT1* and air pollution on neuroprotection, metabolism, and cell survival. Lastly, we currently do not possess epigenetic markers in this cohort to measure gene expression.

Our study has many strengths. First, it is one of the largest population studies by samples size on the role of *SIRT1* in human populations. Second, we had over one decade of follow-up on the survival status of participants. We had a highly diverse group of individuals from within China, including all climatic and geographical regions covering 23 provinces. Third, as our cohort design was initially intended to study socioeconomic determinants of health, we had a wide range of possible confounders to adjust for. Fourth, conducting this study in a developing country, we had high heterogeneity of a range of low and high ambient air pollution levels to see a dose-response relationship. Lastly, a relatively large sample size gave us statistical power for interaction variables and stratified analyses.

## Conclusions

In conclusion, our study suggested a gene-environmental interaction of *SIRT1* genotype and air pollution in a large population-based cohort, provided human epidemiological evidence to validate the many animal studies. If *SIRT1* does protect against PM_2.5_ exposure, those living in areas with high levels of air pollution may benefit from induced *SIRT1* activity through supplementation or interventions. Our findings indicate future clinical trials are needed to validate this hypothesis. If successful, this may be public health and clinical intervention through targeting mechanisms to protect against air pollution insult on the human body.

## Supplementary Information

**Additional file 1.**

## Data Availability

The data that support the findings of this study are available from the corresponding author, upon reasonable request.
