# Supplementary material for: Interaction of Sirtuin 1 (SIRT1) candidate longevity gene and particulate matter (PM2.5) on all-cause mortality: a longitudinal cohort study in China
Source: Environ Health. 2021 Mar 14;20:25. doi: 10.1186/s12940-021-00718-x (PMC7958462; doi:10.1186/s12940-021-00718-x)
Supplement: Supplementary file 1 — Additional file 1. [file 12940_2021_718_MOESM1_ESM.docx]

**PM2.5, *SIRT1* polymorphisms and all-cause mortality among older women and men: the role of gene-environment interaction**

Yao Yao, Linxin Liu, Guang Guo, Yi Zeng, John S. Ji

**Appendix Supplement**

This appendix formed part of the original submission and has been peer reviewed. We post it as supplied by the authors.

**Figure S1………………………………………………………………………………………………………….2**

Derivation of the study population from participants of the Chinese Longitudinal Healthy Longevity Survey (CLHLS).

**Figure S2………………………………………………………………………………………………………….3**

Linkage disequilibrium and haplotype blocks plot of the nine candidate SIRT1 SNPs.

**Table S1……………………………………………………………………………………………………………4**

Candidate SIRT1 SNPs and tag SNPs.

**Table S2. ………………………………………………………………………………………………………….6**

Baseline characteristics of the total study population across different tagging SIRT1 SNPs.

**Table S3……………………………………………………………………………………………………….….9**

Population characteristics of the sample by the inclusion criteria.

**Table S4……………………………………………………………………………………………………….….9**

The interaction between PM2.5 and SIRT1 SNPs (recessive model) on mortality.

**Table S5………………………………………………………………………………………………………….10**

The interaction between PM2.5 and SIRT1 SNPs (dominant model) on mortality.

34,394 participants from 2000-2011 wave

7,083 participants included

Excluded participants aged younger than 65 (545)

Excluded participants without available genetic data (25,220)

Excluded participants not lost in the first follow-up (767)

Excluded non-Han participants and with missing value (567)

Excluded participants without available three-year average PM_2.5_ (212)

**Figure S1.** Derivation of the study population from participants of the Chinese Longitudinal Healthy Longevity Survey (CLHLS).


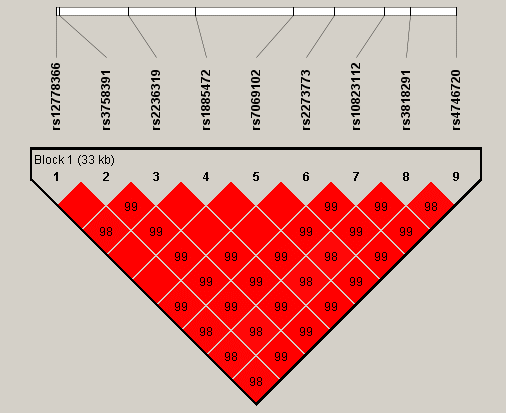


**Figure S2.** Linkage disequilibrium and haplotype blocks plot of the nine candidate SIRT1 SNPs.

All SNPs were in high LD (all D’>0.95).

**Table S1**. Candidate *SIRT1* SNPs and tag SNPs

| SNP | location | Region | Minor allele | Major allele | MAF | Tag SNP | r^2^ |
| --- | --- | --- | --- | --- | --- | --- | --- |
| rs12778366 | Chr10: 69643079 | Upstream | C | T | 0.137 | **rs12778366** | 1 |
| rs3758391 | Chr10: 69643342 | Upstream | C | T | 0.159 | **rs3758391** | 1 |
| rs2236319 | Chr10: 69649220 | Intron | G | A | 0.269 | rs2273773 | 0.996 |
| rs1885472 | Chr10: 69654812 | Intron | C | G | 0.159 | rs3758391 | 0.995 |
| rs7069102 | Chr10: 69663120 | Intron | G | C | 0.158 | rs3758391 | 0.995 |
| rs2273773 | Chr10: 69666598 | Exon 5 | C | T | 0.270 | **rs2273773** | 1 |
| rs10823112 | Chr10: 69670816 | Intron | G | A | 0.270 | rs2273773 | 0.995 |
| rs3818291 | Chr10: 69672999 | Intron | A | G | 0.136 | rs12778366 | 0.975 |
| rs4746720 | Chr10: 69676830 | 3'-UTR | C | T | 0.434 | **rs4746720** | 1 |

Note: The nine candidate SNPs can be represented by four tag SNPs with all r^2^>0.95.

MAF: Minor allele frequency.

SNP selection and Genotyping: Based on our previous CLHLS Genome-Wide Association Study (GWAS)^[[1]](#footnote-1)^, we carried out the extended replication study for 13,228 individuals in CLHLS by using a well-designed and customized chip (Affymetrix Axiom™myDesign™ [384-format]), which targeted about 27,000 longevity-phenotype related Single nucleotide polymorphisms (SNPs). These 27k SNPs include: (a) SNPs associated with longevity and/or health status at old ages identified by us using our GWAS and HRS GWAS datasets; (b) SNPs associated with various chronic diseases including Alzheimer disease, dementia, cognitive and mental problems, and other diseases reported in the literature.

**Table S2.** Baseline characteristics of the total study population across different tagging *SIRT1* SNPs

|  | **Overall**  **(n=7083)** | ***SIRT1*_391** | | |  | ***SIRT1_*366** | | |  | ***SIRT1*_773** | | |  | ***SIRT1*_720** | | |
| --- | --- | --- | --- | --- | --- | --- | --- | --- | --- | --- | --- | --- | --- | --- | --- | --- |
|  |  | **0 (n=5006)** | **1 (n=1887)** | **2 (n=184)** |  | **0 (n=5281)** | **1 (n=1635)** | **2 (n=139)** |  | **0 (n=3795)** | **1 (n=2743)** | **2 (n=533)** |  | **0 (n=2292)** | **1 (n=3383)** | **2 (n=1371)** |
| **Survival time (years): Mean (SD)** | 6.05 (3.54) | 6.07 (3.54) | 5.97 (3.55) | 6.04 (3.38) |  | 6.05 (3.54) | 6.06 (3.54) | 5.57 (3.10) |  | 6.04 (3.50) | 6.04 (3.59) | 6.14 (3.54) |  | 6.02 (3.52) | 6.03 (3.57) | 6.10 (3.49) |
| **Three-year average PM_2.5_ (µg/m³): Mean (SD)** | 51.0 (13.5) | 50.8 (13.5) | 51.3 (13.4) | 52.4 (14.5) |  | 51.4 (13.4) | 49.9 (13.6) | 47.6 (13.4) |  | 51.1 (13.4) | 50.6 (13.5) | 52.2 (14.0) |  | 50.8 (13.9) | 50.7 (13.3) | 51.7 (13.1) |
| **Wave: n (%)** |  |  |  |  |  |  |  |  |  |  |  |  |  |  |  |  |
| 2000 | 204 (2.9) | 145 (2.9) | 56 (3.0) | 2 (1.1) |  | 155 (2.9%) | 47 (2.9%) | 2 (1.4%) |  | 103 (2.7%) | 88 (3.2%) | 13 (2.4%) |  | 60 (2.6) | 104 (3.1) | 39 (2.8) |
| 2002 | 1670 (23.6) | 1185 (23.7) | 437 (23.2) | 47 (25.5) |  | 1248 (23.6%) | 392 (24.0%) | 19 (13.7%) |  | 882 (23.2%) | 653 (23.8%) | 130 (24.4%) |  | 539 (23.5) | 792 (23.4) | 325 (23.7) |
| 2005 | 1377 (19.4) | 983 (19.6) | 361 (19.1) | 32 (17.4) |  | 1034 (19.6%) | 303 (18.5%) | 35 (25.2%) |  | 754 (19.9%) | 515 (18.8%) | 107 (20.1%) |  | 442 (19.3) | 637 (18.8) | 291 (21.2) |
| 2008 | 3081 (43.5) | 2160 (43.1) | 837 (44.4) | 81 (44.0) |  | 2273 (43.0%) | 732 (44.8%) | 68 (48.9%) |  | 1647 (43.4%) | 1208 (44.0%) | 222 (41.7%) |  | 1008 (44.0) | 1495 (44.2) | 563 (41.1) |
| 2011 | 751 (10.6) | 533 (10.6) | 196 (10.4) | 22 (12.0) |  | 571 (10.8%) | 161 (9.8%) | 15 (10.8%) |  | 409 (10.8%) | 279 (10.2%) | 61 (11.4%) |  | 243 (10.6) | 355 (10.5) | 153 (11.2) |
| **Sex: n (%)** |  |  |  |  |  |  |  |  |  |  |  |  |  |  |  |  |
| Male | 3406 (48.1) | 2394 (47.8) | 917 (48.6) | 92 (50.0) |  | 2524 (47.8%) | 806 (49.3%) | 61 (43.9%) |  | 1833 (48.3%) | 1319 (48.1%) | 248 (46.5%) |  | 1083 (47.3) | 1672 (49.4) | 631 (46.0) |
| Female | 3677 (51.9) | 2612 (52.2) | 970 (51.4) | 92 (50.0) |  | 2757 (52.2%) | 829 (50.7%) | 78 (56.1%) |  | 1962 (51.7%) | 1424 (51.9%) | 285 (53.5%) |  | 1209 (52.7) | 1711 (50.6) | 740 (54.0) |
| **Age(year): Mean (SD)** | 81.1 (11.5) | 80.9 (11.5) | 81.5 (11.6) | 81.9 (11.7) |  | 81.2 (11.6) | 80.8 (11.5) | 79.3 (10.4) |  | 81.0 (11.4) | 81.4 (11.7) | 80.2 (11.4) |  | 81.1 (11.5) | 81.1 (11.6) | 81.1 (11.3) |
| **Age group: n (%)** |  |  |  |  |  |  |  |  |  |  |  |  |  |  |  |  |
| 65-79 | 3271 (46.2) | 2346 (46.9) | 838 (44.4) | 85 (46.2) |  | 2415 (45.7%) | 778 (47.6%) | 69 (49.6%) |  | 1752 (46.2%) | 1245 (45.4%) | 270 (50.7%) |  | 1075 (46.9) | 1552 (45.9) | 623 (45.4) |
| 80-89 | 1840 (26.0) | 1300 (26.0) | 489 (25.9) | 48 (26.1) |  | 1369 (25.9%) | 416 (25.4%) | 46 (33.1%) |  | 1011 (26.6%) | 689 (25.1%) | 136 (25.5%) |  | 596 (26.0) | 859 (25.4) | 375 (27.4) |
| 90-99 | 1305 (18.4) | 905 (18.1) | 369 (19.6) | 31 (16.8) |  | 986 (18.7%) | 292 (17.9%) | 20 (14.4%) |  | 695 (18.3%) | 529 (19.3%) | 78 (14.6%) |  | 404 (17.6) | 640 (18.9) | 257 (18.7) |
| 100+ | 667 (9.4) | 455 (9.1) | 191 (10.1) | 20 (10.9) |  | 511 (9.7%) | 149 (9.1%) | 4 (2.9%) |  | 337 (8.9%) | 280 (10.2%) | 49 (9.2%) |  | 217 (9.5) | 332 (9.8) | 116 (8.5) |
| **Education(year): Mean (SD)** | 2.38 (3.59) | 2.40 (3.61) | 2.35 (3.53) | 2.38 (3.60) |  | 2.35 (3.55) | 2.48 (3.68) | 2.45 (3.64) |  | 2.41 (3.60) | 2.37 (3.59) | 2.22 (3.44) |  | 2.33 (3.55) | 2.41 (3.59) | 2.37 (3.60) |
| **Education group: n (%)** | |  |  |  |  |  |  |  |  |  |  |  |  |  |  |  |
| 0 year | 4045 (57.1) | 2858 (57.1) | 1078 (57.1) | 105 (57.1) |  | 3029 (57.4%) | 918 (56.1%) | 81 (58.3%) |  | 2150 (56.7%) | 1577 (57.5%) | 311 (58.3%) |  | 1323 (57.7) | 1919 (56.7) | 785 (57.3) |
| 1-6 years | 2211 (31.2) | 1557 (31.1) | 592 (31.4) | 61 (33.2) |  | 1649 (31.2%) | 518 (31.7%) | 39 (28.1%) |  | 1195 (31.5%) | 846 (30.8%) | 167 (31.3%) |  | 713 (31.1) | 1061 (31.4) | 428 (31.2) |
| >6 years | 827 (11.7) | 591 (11.8) | 217 (11.5) | 18 (9.8) |  | 603 (11.4%) | 199 (12.2%) | 19 (13.7%) |  | 450 (11.9%) | 320 (11.7%) | 55 (10.3%) |  | 256 (11.2) | 403 (11.9) | 158 (11.5) |
| **Residence: n (%)** |  |  |  |  |  |  |  |  |  |  |  |  |  |  |  |  |
| Urban | 2358 (33.3) | 1693 (33.8) | 597 (31.6) | 66 (35.9) |  | 1759 (33.3%) | 548 (33.5%) | 42 (30.2%) |  | 1265 (33.3%) | 908 (33.1%) | 181 (34.0%) |  | 741 (32.3) | 1144 (33.8) | 460 (33.6) |
| Rural | 4725 (66.7) | 3313 (66.2) | 1290 (68.4) | 118 (64.1) |  | 3522 (66.7%) | 1087 (66.5%) | 97 (69.8%) |  | 2530 (66.7%) | 1835 (66.9%) | 352 (66.0%) |  | 1551 (67.7) | 2239 (66.2) | 911 (66.4) |
| **Marriage: n (%)** |  |  |  |  |  |  |  |  |  |  |  |  |  |  |  |  |
| Married | 3198 (45.2) | 2307 (46.1) | 811 (43.0) | 79 (42.9) |  | 2365 (44.8%) | 751 (45.9%) | 72 (51.8%) |  | 1731 (45.6%) | 1215 (44.3%) | 247 (46.3%) |  | 1023 (44.6) | 1531 (45.3) | 628 (45.8) |
| Not married | 3885 (54.8) | 2699 (53.9) | 1076 (57.0) | 105 (57.1) |  | 2916 (55.2%) | 884 (54.1%) | 67 (48.2%) |  | 2064 (54.4%) | 1528 (55.7%) | 286 (53.7%) |  | 1269 (55.4) | 1852 (54.7) | 743 (54.2) |
| **Occupation: n (%)** |  |  |  |  |  |  |  |  |  |  |  |  |  |  |  |  |
| non-manual | 558 (7.9) | 397 (7.9) | 142 (7.5) | 18 (9.8) |  | 415 (7.9%) | 132 (8.1%) | 8 (5.8%) |  | 310 (8.2%) | 210 (7.7%) | 36 (6.8%) |  | 176 (7.7) | 259 (7.7) | 119 (8.7) |
| manual | 6525 (92.1) | 4609 (92.1) | 1745 (92.5) | 166 (90.2) |  | 4866 (92.1%) | 1503 (91.9%) | 131 (94.2%) |  | 3485 (91.8%) | 2533 (92.3%) | 497 (93.2%) |  | 2116 (92.3) | 3124 (92.3) | 1252 (91.3) |
| **Exercise: n (%)** |  |  |  |  |  |  |  |  |  |  |  |  |  |  |  |  |
| Current | 2213 (31.2) | 1606 (32.1) | 557 (29.5) | 48 (26.1) |  | 1625 (30.8%) | 544 (33.3%) | 34 (24.5%) |  | 1143 (30.1%) | 888 (32.4%) | 179 (33.6%) |  | 720 (31.4) | 1063 (31.4) | 416 (30.3) |
| Former | 304 (4.3) | 214 (4.3) | 81 (4.3) | 8 (4.3) |  | 236 (4.5%) | 59 (3.6%) | 9 (6.5%) |  | 171 (4.5%) | 111 (4.0%) | 22 (4.1%) |  | 92 (4.0) | 145 (4.3) | 66 (4.8) |
| Never | 4566 (64.5) | 3186 (63.6) | 1249 (66.2) | 128 (69.6) |  | 3420 (64.8%) | 1032 (63.1%) | 96 (69.1%) |  | 2481 (65.4%) | 1744 (63.6%) | 332 (62.3%) |  | 1480 (64.6) | 2175 (64.3) | 889 (64.8) |
| **Smoking: n (%)** |  |  |  |  |  |  |  |  |  |  |  |  |  |  |  |  |
| Current | 1617 (22.8) | 1137 (22.7) | 432 (22.9) | 47 (25.5) |  | 1222 (23.1%) | 358 (21.9%) | 28 (20.1%) |  | 866 (22.8%) | 632 (23.0%) | 118 (22.1%) |  | 520 (22.7) | 766 (22.6) | 320 (23.3) |
| Former | 933 (13.2) | 637 (12.7) | 270 (14.3) | 26 (14.1) |  | 680 (12.9%) | 234 (14.3%) | 15 (10.8%) |  | 518 (13.6%) | 349 (12.7%) | 63 (11.8%) |  | 301 (13.1) | 448 (13.2) | 178 (13.0) |
| Never | 4533 (64.0) | 3232 (64.6) | 1185 (62.8) | 111 (60.3) |  | 3379 (64.0%) | 1043 (63.8%) | 96 (69.1%) |  | 2411 (63.5%) | 1762 (64.2%) | 352 (66.0%) |  | 1471 (64.2) | 2169 (64.1) | 873 (63.7) |
| **Alcohol: n (%)** |  |  |  |  |  |  |  |  |  |  |  |  |  |  |  |  |
| Current | 1619 (22.9) | 1120 (22.4) | 447 (23.7) | 50 (27.2) |  | 1187 (22.5%) | 393 (24.0%) | 28 (20.1%) |  | 881 (23.2%) | 628 (22.9%) | 110 (20.6%) |  | 537 (23.4) | 769 (22.7) | 306 (22.3) |
| Former | 612 (8.6) | 436 (8.7) | 159 (8.4) | 16 (8.7) |  | 439 (8.3%) | 157 (9.6%) | 14 (10.1%) |  | 354 (9.3%) | 216 (7.9%) | 40 (7.5%) |  | 185 (8.1) | 296 (8.7) | 127 (9.3) |
| Never | 4852 (68.5) | 3450 (68.9) | 1281 (67.9) | 118 (64.1) |  | 3655 (69.2%) | 1085 (66.4%) | 97 (69.8%) |  | 2560 (67.5%) | 1899 (69.2%) | 383 (71.9%) |  | 1570 (68.5) | 2318 (68.5) | 938 (68.4) |

Table S3. Population characteristics of the sample by the inclusion criteria

|  | **CLHLS 2000-2011 wave 33849 participants aged >=65** | | | | | | | | |
| --- | --- | --- | --- | --- | --- | --- | --- | --- | --- |
|  | **Overall (n=33849)** | **Without genetic data (N=25220)** | **With genetic data (N=8629)** | | | | | | |
|  |  |  | **Overall (N=8629)** | **Lost in the first follow-up (N=767)** | **Not lost in the first follow-up (N=7862)** | | | | |
|  |  |  |  |  | **Overall (N=7862)** | **Non-Han or with missing value in covariates (N=567)** | **Han and without missing value in covariates(N=7295)** | | |
|  |  |  |  |  |  |  | **Overall(N=7295)** | **PM2.5 missing (N=212)** | **Final sample PM2.5 not missing (N=7083)** |
| **Gender** |  |  |  |  |  |  |  |  |  |
| Male | 13938 (41.2%) | 9825 (39.0%) | 4113 (47.7%) | 363 (47.3%) | 3750 (47.7%) | 253 (44.6%) | 3497 (47.9%) | 91 (42.9%) | 3406 (48.1%) |
| Female | 19911 (58.8%) | 15395 (61.0%) | 4516 (52.3%) | 404 (52.7%) | 4112 (52.3%) | 314 (55.4%) | 3798 (52.1%) | 121 (57.1%) | 3677 (51.9%) |
| **Age(year): Mean (SD)** | 87.5 (11.7) | 89.5 (11.1) | 81.7 (11.7) | 86.0 (11.7) | 81.3 (11.6) | 82.6 (12.1) | 81.2 (11.5) | 86.1 (11.1) | 81.1 (11.5) |
| **Education year** |  |  |  |  |  |  |  |  |  |
| Mean (SD) | 1.87 (3.34) | 1.70 (3.24) | 2.35 (3.57) | 2.39 (3.65) | 2.34 (3.56) | 2.06 (3.30) | 2.36 (3.57) | 1.69 (3.09) | 2.38 (3.59) |
| Missing | 203 (0.6%) | 178 (0.7%) | 25 (0.3%) | 5 (0.7%) | 20 (0.3%) | 20 (3.5%) | 0 (0%) | / | / |
| **Residence** |  |  |  |  |  |  |  |  |  |
| City/Town | 14656 (43.3%) | 11711 (46.4%) | 2945 (34.1%) | 354 (46.2%) | 2591 (33.0%) | 154 (27.2%) | 2437 (33.4%) | 79 (37.3%) | 2358 (33.3%) |
| Rural | 19193 (56.7%) | 13509 (53.6%) | 5684 (65.9%) | 413 (53.8%) | 5271 (67.0%) | 413 (72.8%) | 4858 (66.6%) | 133 (62.7%) | 4725 (66.7%) |
| **Marriage** |  |  |  |  |  |  |  |  |  |
| Currently married | 9547 (28.2%) | 5813 (23.0%) | 3734 (43.3%) | 263 (34.3%) | 3471 (44.1%) | 192 (33.9%) | 3279 (44.9%) | 81 (38.2%) | 3198 (45.2%) |
| Not currently married | 24295 (71.8%) | 19404 (76.9%) | 4891 (56.7%) | 503 (65.6%) | 4388 (55.8%) | 372 (65.6%) | 4016 (55.1%) | 131 (61.8%) | 3885 (54.8%) |
| Missing | 7 (0.0%) | 3 (0.0%) | 4 (0.0%) | 1 (0.1%) | 3 (0.0%) | 3 (0.5%) | 0 (0%) | / | / |
| **Exercise** |  |  |  |  |  |  |  |  |  |
| Current | 9396 (27.8%) | 6749 (26.8%) | 2647 (30.7%) | 244 (31.8%) | 2403 (30.6%) | 101 (17.8%) | 2302 (31.6%) | 89 (42.0%) | 2213 (31.2%) |
| Former | 2532 (7.5%) | 2086 (8.3%) | 446 (5.2%) | 78 (10.2%) | 368 (4.7%) | 28 (4.9%) | 340 (4.7%) | 36 (17.0%) | 304 (4.3%) |
| Never | 21857 (64.6%) | 16351 (64.8%) | 5506 (63.8%) | 439 (57.2%) | 5067 (64.4%) | 414 (73.0%) | 4653 (63.8%) | 87 (41.0%) | 4566 (64.5%) |
| Missing | 64 (0.2%) | 34 (0.1%) | 30 (0.3%) | 6 (0.8%) | 24 (0.3%) | 24 (4.2%) | 0 (0%) | / | / |
| **Smoking** |  |  |  |  |  |  |  |  |  |
| Current | 6116 (18.1%) | 4217 (16.7%) | 1899 (22.0%) | 131 (17.1%) | 1768 (22.5%) | 115 (20.3%) | 1653 (22.7%) | 36 (17.0%) | 1617 (22.8%) |
| Former | 4588 (13.6%) | 3491 (13.8%) | 1097 (12.7%) | 95 (12.4%) | 1002 (12.7%) | 39 (6.9%) | 963 (13.2%) | 30 (14.2%) | 933 (13.2%) |
| Never | 23118 (68.3%) | 17490 (69.3%) | 5628 (65.2%) | 540 (70.4%) | 5088 (64.7%) | 409 (72.1%) | 4679 (64.1%) | 146 (68.9%) | 4533 (64.0%) |
| Missing | 27 (0.1%) | 22 (0.1%) | 5 (0.1%) | 1 (0.1%) | 4 (0.1%) | 4 (0.7%) | 0 (0%) | / | / |
| **Drinking alcohol** |  |  |  |  |  |  |  |  |  |
| Current | 6471 (19.1%) | 4593 (18.2%) | 1878 (21.8%) | 129 (16.8%) | 1749 (22.2%) | 103 (18.2%) | 1646 (22.6%) | 27 (12.7%) | 1619 (22.9%) |
| Former | 3337 (9.9%) | 2606 (10.3%) | 731 (8.5%) | 66 (8.6%) | 665 (8.5%) | 35 (6.2%) | 630 (8.6%) | 18 (8.5%) | 612 (8.6%) |
| Never | 24007 (70.9%) | 17996 (71.4%) | 6011 (69.7%) | 571 (74.4%) | 5440 (69.2%) | 421 (74.3%) | 5019 (68.8%) | 167 (78.8%) | 4852 (68.5%) |
| Missing | 34 (0.1%) | 25 (0.1%) | 9 (0.1%) | 1 (0.1%) | 8 (0.1%) | 8 (1.4%) | 0 (0%) | / | / |

**Table S4.** The interaction between PM2.5 and *SIRT1* SNPs (recessive model) on mortality

| Recessive model | Model without interaction term | |  | Model with interaction term | | |
| --- | --- | --- | --- | --- | --- | --- |
|  | HR (95% CI) | *p*-value |  | β | SE | *p*-value |
| **Carrying *SIRT1*_366 minor allele status** |  |  |  |  |  |  |
| 0 or 1 copy | Ref | / |  | / | / | / |
| 2 copies | 1.227 (0.919,1.639) | 0.164 |  | -0.051 | 0.543 | 0.926 |
| **10-μg/m^3^ unit of PM_2.5_** | 1.083 (1.054,1.112) | 8.72E-09 |  | 0.079 | 0.014 | 1.67E-08 |
| **Interaction term** |  |  |  |  |  |  |
| zero or one SIRT1_366 minor allele * PM_2.5_ | / | / |  | / | / | / |
| two SIRT1_366 minor allele * PM_2.5_ | / | / |  | 0.052 | 0.105 | 0.622 |
|  |  |  |  |  |  |  |
| **Carrying *SIRT1_391* minor allele status** |  |  |  |  |  |  |
| 0 or 1 copy | Ref | / |  | / | / | / |
| 2 copies | 0.763 (0.598,0.972) | 0.029 |  | -1.382 | 0.575 | 0.016 |
| **10-μg/m^3^ unit of PM_2.5_** | 1.082 (1.053,1.112) | 1.11E-08 |  | 0.075 | 0.014 | 8.02E-08 |
| **Interaction term** |  |  |  |  |  |  |
| zero or one SIRT1_391 minor allele * PM_2.5_ | / | / |  | / | / | / |
| two SIRT1_391 minor allele * PM_2.5_ | / | / |  | 0.206 | 0.101 | 0.042 |
|  |  |  |  |  |  |  |
| **Carrying *SIRT1_773* minor allele status** |  |  |  |  |  |  |
| 0 or 1 copy | Ref | / |  | / | / | / |
| 2 copies | 0.95 (0.82,1.102) | 0.5 |  | -0.427 | 0.31 | 0.168 |
| **10-μg/m^3^ unit of PM_2.5_** | 1.082 (1.053,1.112) | 1.17E-08 |  | 0.074 | 0.014 | 2.31E-07 |
| **Interaction term** |  |  |  |  |  |  |
| zero or one SIRT1_773 minor allele * PM_2.5_ | / | / |  | / | / | / |
| two SIRT1_773 minor allele * PM_2.5_ | / | / |  | 0.069 | 0.055 | 0.206 |
|  |  |  |  |  |  |  |
| **Carrying *SIRT1_720* minor allele status** |  |  |  |  |  |  |
| 0 or 1 copy | Ref | / |  | / | / | / |
| 2 copies | 0.975 (0.888,1.071) | 0.599 |  | -0.031 | 0.197 | 0.876 |
| **10-μg/m^3^ unit of PM_2.5_** | 1.081 (1.052,1.111) | 1.86E-08 |  | 0.078 | 0.015 | 3.38E-07 |
| **Interaction term** |  |  |  |  |  |  |
| zero or one SIRT1_720 minor allele * PM_2.5_ | / | / |  | / | / | / |
| two SIRT1_720 minor allele * PM_2.5_ | / | / |  | 0.001 | 0.036 | 0.976 |

*All models were adjusted for age at baseline, sex, education, marriage, occupation, residence, exercise, smoking, and alcohol consumption.

**Table S5.** The interaction between PM_2.5_ and *SIRT1* SNPs (dominant model) on mortality

| Dominant model | Model without interaction term | |  | Model with interaction term | | |
| --- | --- | --- | --- | --- | --- | --- |
|  | HR (95% CI) | *p*-value |  | β | SE | *p*-value |
| **Carrying *SIRT1*_366 minor allele status** |  |  |  |  |  |  |
| 0 copy | Ref | / |  | / | / | / |
| 1 or 2 copies | 1.069 (0.982,1.164) | 0.124 |  | 0.237 | 0.167 | 0.155 |
| **10-μg/m^3^ unit of PM_2.5_** | 1.083 (1.054,1.113) | 8.05E-09 |  | 0.088 | 0.016 | 3.51E-08 |
| **Interaction term** |  |  |  |  |  |  |
| zero *SIRT1*_366 minor allele * PM_2.5_ | / | / |  | / | / | / |
| one or two *SIRT1*_366 minor allele * PM_2.5_ | / | / |  | -0.033 | 0.031 | 0.292 |
|  |  |  |  |  |  |  |
| **Carrying *SIRT1*_391 minor allele status** |  |  |  |  |  |  |
| 0 copy | Ref | / |  | / | / | / |
| 1 or 2 copies | 0.999 (0.922,1.083) | 0.989 |  | -0.338 | 0.163 | 0.039 |
| **10-μg/m^3^ unit of PM_2.5_** | 1.082 (1.053,1.112) | 1.16E-08 |  | 0.06 | 0.016 | 2.66E-04 |
| **Interaction term** |  |  |  |  |  |  |
| zero *SIRT1*_391 minor allele * PM_2.5_ | / | / |  | / | / | / |
| one or two *SIRT1*_391 minor allele * PM_2.5_ | / | / |  | 0.064 | 0.03 | 0.032 |
|  |  |  |  |  |  |  |
| **Carrying *SIRT1*_773 minor allele status** |  |  |  |  |  |  |
| 0 copy | Ref | / |  | / | / | / |
| 1 or 2 copies | 0.993 (0.922,1.069) | 0.857 |  | 0.046 | 0.148 | 0.755 |
| **10-μg/m^3^ unit of PM_2.5_** | 1.082 (1.053,1.111) | 1.34E-08 |  | 0.083 | 0.019 | 1.32E-05 |
| **Interaction term** |  |  |  |  |  |  |
| zero *SIRT1*_773 minor allele * PM_2.5_ | / | / |  | / | / | / |
| one or two *SIRT1*_773 minor allele * PM_2.5_ | / | / |  | -0.01 | 0.027 | 0.711 |
|  |  |  |  |  |  |  |
| **Carrying *SIRT1*_720 minor allele status** |  |  |  |  |  |  |
| 0 copy | Ref | / |  | / | / | / |
| 1 or 2 copies | 1.031 (0.953,1.116) | 0.448 |  | 0.337 | 0.159 | 0.034 |
| **10-μg/m^3^ unit of PM_2.5_** | 1.080 (1.051,1.110) | 2.27E-08 |  | 0.117 | 0.024 | 1.44E-06 |
| **Interaction term** |  |  |  |  |  |  |
| zero *SIRT1*_720 minor allele * PM_2.5_ | / | / |  | / | / | / |
| one or two *SIRT1*_720 minor allele * PM_2.5_ | / | / |  | -0.059 | 0.029 | 0.046 |

*All models were adjusted for age at baseline, sex, education, marriage, occupation, residence, exercise, smoking, and alcohol consumption.

1. Zeng Y, Nie C, Min J, et al. Novel loci and pathways significantly associated with longevity. *Sci Rep*. 2016;6:21243. Published 2016 Feb 25. doi:10.1038/srep21243 [↑](#footnote-ref-1)
